# Less car, more bicycle? Generation Y as pioneers of changing everyday mobility in Germany

**DOI:** 10.1186/s12544-023-00575-4

**Published:** 2023-01-30

**Authors:** Dirk Konietzka, Lukas Neugebauer

**Affiliations:** Institute of Sociology, TU Braunschweig, Bienroder Weg 97, 38106 Braunschweig, Germany

**Keywords:** Car use, Bicycle use, Birth cohorts, Generation Y, Generation X, Time series, Metropolises, Rural areas, Germany

## Abstract

**Background:**

The paper examines whether the widespread assumption holds that younger birth cohorts (referred to as Millennials or Generation Y) act as pioneers of changing everyday mobility.

**Methodology:**

Based on the time-series dataset "Mobility in Germany" (Mobilität in Deutschland), cohort-specific changes in everyday bicycle and car use that have occurred between 2002 and 2017 are analyzed. The empirical analyses are differentiated by age-group and settlement type. Additionally, socio-structural factors are taken into account.

**Results:**

The results show a decline in the predominant everyday use of cars in metropolitan cities, especially among Generation Y. However, the Millennials do not emerge as pioneers of the trend toward predominant bicycle use. The results challenge the assumption that changes in everyday mobility are essentially driven by generational change.

## Introduction

In recent years, the mobility behavior of younger birth cohorts has attracted the interest of transportation research (cf. [1–5]). The so-called Millennial generation—i.e., those born between 1980 and 2000—are considered to be particularly environmentally friendly [6, 7], and to prefer bicycles [8, 9] as well as multi- or intermodal modes of traveling [10–12] as means of transportation. Changing mobility attitudes and behaviors among the younger cohorts are also reflected in decreasing rates of car ownership ([13]: 38), a declining subjective relevance of the car, and the erosion of the car as a status symbol [14, 15].

However, despite the interest in younger cohorts’ mobility behavior, few empirical studies have analyzed cohort-specific mobility trends. The existing studies have either looked at selected age groups [16] or considered different cohorts in a cross-sectional design [5]. Given that cross-sectional analyses cannot disentangle age and cohort effects, they leave open the question of whether young people's mobility behavior is attributable to *cohort change,* or merely to *age-specific mobility patterns*. Against this background, our paper differs from the existing literature *by comparing cohort-specific dynamics in everyday bicycle and car use*.

From a theoretical point of view, cohort-specific changes in mobility behavior may result from different processes, including changes in attitudes and values [17], as well as in the social composition of subsequent cohorts, their resource endowments, or (infra)structural conditions. Given that mobility needs vary significantly between biographical stages, shifting life course patterns may explain some of the changes in everyday mobility. For example, the life courses of younger cohorts have been shaped by prolonged educational participation and the postponement of both labor force entry and family formation [18, 19]. Younger cohorts have also experienced less comfortable employment and income conditions than preceding generations [20]. Moreover, empirical studies have identified socio-structural differences in mobility behavior. Bicycle use is most widespread among the higher educated [21] and specific social milieus [22]. Since younger and more educated people are also more likely to live in large cities and metropolitan areas [23﻿, 24, 25], their transport choices may be influenced by urban infrastructure, such as bicycle path networks. Thus, the trends toward higher education and the influx of more educated people into metropolitan areas may at least partially explain the recent changes in cohort-specific transport behavior. In a similar vein, the shifts in employment status and life course patterns in early adulthood may have influenced individual mobility demands. Against this background, the paper’s second aim is to assess *whether cohort differences in everyday mobility are due to changes in the structural composition* of the cohorts under consideration.

Finally, given that the existing literature has emphasized the role of local opportunity structures in everyday mobility, we specifically investigate how cohort-specific trends in bicycling and automobility *are diverging between settlement types*. Our main focus is on advanced “post-industrial” metropolises that are considered to be sustainable urban lifestyle hotspots, and that are especially attractive to the younger and higher educated social strata [26, 27]. We therefore suspect that trends in bicycle mobility are particularly visible among the younger cohorts living in the post-industrial metropolises.

For our analyses, we use the time-series dataset "Mobility in Germany" (*Zeitreihendatensatz Mobilität in Deutschland (MiD)),* which covers the period from 2002 to 2017, and allows us to investigate both age- and cohort-specific behavioral changes over a 15-year period [13]. We specifically investigate whether the Millennials (or members of Generation Y) use *cars less frequently and bicycles more frequently* in their everyday lives than predecessor cohorts. The analyses are differentiated by cohort, age, settlement type, and socio-structural factors. In the following section, we will develop our conceptual framework of travel mode choice, and outline the current state of the research (Sect. 2). After describing our data and methods (Sect. 3), we will present our findings (Sect. 4). Finally, we will summarize and reflect on our key findings (Sect. 5).

## Conceptual framework and previous research

People's everyday mobility behavior is influenced by a wide array of factors, including their socioeconomic resources, opportunity structures, and life course-specific preferences. Furthermore, everyday mobility is affected by cohort-specific values and attitudes toward modes of transportation. In this sense, the process of cohort change—i.e., the successive replacement of older by younger cohorts—may be considered a basic mechanism of change in everyday mobility [2]. To measure everyday mobility, researchers have used a range of indicators, from symbolic meaning and subjective importance, to ownership and availability, to the actual use of cars and bicycles. The latter studies examined, for example, total travel distances (in kilometers or miles) and travel time (in minutes) on a reference date (e.g., [28, 29],commuting distances to work [30, 31],frequency of car use per week [12]; and driver's license ownership [11, 16, 17, 32, ]). While these characteristics are correlated with each other, they measure different aspects of everyday mobility. Not surprisingly, the results of these analyses were often conflicting, or were not directly comparable. In the following, we summarize key findings from the research literature.

Analyses based on the MiD time-series dataset reported that in Germany, the daily volume of car use decreased by 6 percent between 2002 and 2017 [13]: 10). The number of daily trips declined slightly from an average of 3.3 trips per day to an average of 3.1 trips per day, regardless of the mode of transport. In contrast, the mean daily distance traveled increased from 33 to 39 km (ibid.). In terms of the modal split of the transport volume (the distances traveled), the share of the study population using motorized individual transport fell significantly (from 52 to 43 percent) in the 20–29 age group, and decreased slightly (from 55 to 52%) in the 30–39 age group. However, this share remained unaltered or increased among the older age groups [13]: 51). The "Mobility of Young People" (“Mobilität junger Menschen”) study found that starting in the early 1990s, the share of young adults in Germany who were using a car for daily trips was declining, while the share who were using public transport was increasing. These changes were attributed to greater proportions of the population who are in education, residing in urban areas and living in single-person households, as well as to a decline in income among young adults [10]: 5). It has also been shown that car ownership in the 18–34 age group increases with income, employment, and living in a multi-person household (ibid.),while it decreases with tertiary education and living in a female-headed household. Kuhnimhof/Armoogum et al. [16] and [11] also found a decline in daily car use and car ownership among the 18–34 age group, which they attributed to changes in household composition, employment patterns, and residential location. The decrease in car use was compensated for by the use of other means of transport, especially among men (ibid.). Schleiffer et al. [33] observed that while students place a high value on private car ownership, their life course-related characteristics (household and living arrangements, employment, etc.), as well as their personal interests and attitudes, tend to delay their transition to car ownership.

International studies have reported similar findings. A decline in car use among young people has, for example, been reported for the Netherlands [20] and the United States [34]. Grimal [5]: 22 found that the mobility behavior of Millennials in France has diverged from that of older generations. He attributed these patterns to biographical factors, in particular to the postponement of entry into working life and family formation, as well as to changes in lifestyles and economic conditions. An association between temporal shifts in life stages and reduced car use has also been observed by Metz [35] and Delbosc and Currie [1]. As explanations for the decline in car use among young adults, Davis et al. [34] and Van den Waard et al. [20] pointed to the worsened economic situation of young people, Hjortol [24], Oakil et al. [25] highlighted the increasing concentration of young people in densely populated urban areas (cf. [16, 17], while Van den Waard et al. [20] emphasized the expansion of public transport infrastructure in densely populated areas. Finally, some studies have noted the changing attitudes of young people toward the automobile. There is, for example, evidence that younger age groups no longer see the car as a status symbol ([1, 17]), and that their environmental awareness has increased [36, 37].

With regard to bicycle use, the literature has reported partially opposing trends. The MiD time-series showed a slight rise in bicycle use (modal split) in all age groups, and an increase from 7 to 11% between 2002 and 2017, specifically in the 20–29 age group [13]: 51). The share of bicycle travel increased by 13% across all age groups (ibid.: 10). However, bicycle ownership increased in metropolitan areas only, while it decreased in rural areas (ibid.). A study by Lanzendorf et al. [38] based on the MiD's trips dataset for 2002 and 2008 also found that the number of daily trips made by bicycle had increased in the metropolitan areas of Berlin, Frankfurt, Hamburg, and Munich. In a similar vein, Hudde [39] reported that bicycle use increased in Germany between 1996 and 2018, but also noted that this “cycling boom” was largely among higher educated individuals in medium-sized and large cities [39]. The analysis, which was based on the German Mobility Panel (MOP), showed that in 2018, highly educated individuals in medium-sized and large cities used bicycles on average for more minutes than lower educated individuals. The findings also indicated that in rural areas, the volumes and differences were smaller [39]: 6. Furthermore, an analysis of the MiD travel dataset 2017 showed that in cities with more than 50,000 inhabitants, higher educated working individuals were systematically more likely to use a bicycle than lower educated individuals [21]. The author concluded that for the higher educated, environmental values, health awareness, and the view of cycling as a sustainable “lifestyle” were crucial factors in their bicycle use.

In a similar vein, international studies identified socio-structural differences in bicycle use. According to a Swedish study [40], the probability of cycling or walking is inversely related to income. There is also evidence that women are less likely to use a bicycle than men, and that psychological factors (such as environmental and health awareness) influence both cycling and walking. Emond et al. [41] reported differences in bicycle use by education and gender for several cities in the western United States. In addition to gender differences in socialization processes and social attributions, men and women differ with respect to their perceptions of safety, which again points to the role of transportation infrastructure in the propensity to use a bicycle among diverse social groups.

Taken together, these findings suggest that whether individuals are inclined to use a car or a bicycle as a transport mode is determined by various factors, most notably by residential location, infrastructure [23], life course events (including [1, 31, 42, 43]), education, income, and commuting distance [40, 44]. However, the existing body of research has provided little evidence on the *cohort-specific changes in mobility behavior and their socio-structural underpinnings,* and, in particular, on the changing composition of birth cohorts and the differences between distinct settlement types.

In the following, we will empirically assess for the German case cohort-specific changes in everyday bicycle and car use. As an indicator, we use the "(almost) daily frequency of use" of bicycles and cars. Measurements that address *regular* bicycle and car use tend to find a higher degree of usage than studies that focus on the share of bicycles in traffic volume measured on a reference date [23]: 29). However, our indicator is less dependent on day of the week, seasons, weather, and local geography (ibid.). Thus, data on *regular bicycle and car use* provide “more generalized” information about the importance of both means of transportation within the realm of people's everyday mobility. In our empirical analyses, we will use a *combined indicator* of the frequency of individual bicycle use *and* automobility, which takes into account that bicycle and automobile use can substitute or complement each other, and that these patterns may vary between social groups and cohorts. We are specifically interested in assessing to what degree the younger cohorts use bicycles, and not cars, as their *primary mode* of transportation (for more details, see Sect. 3).

Based on previous studies that reported an increase in the frequency of everyday bicycle use and a decrease in car use in Germany between 2002 and 2017, we expect to find a decline in predominant car use (hypothesis 1a) and an increase in predominant bicycle use (hypothesis 1b). These trends are supposed to be most pronounced among the younger birth cohorts (Generation Y).

At the same time, the existing body of research has suggested that cohort-specific changes in mobility behavior have been particularly large in metropolitan areas, while the changes in car and bicycle use have been more moderate in smaller towns and rural regions. We therefore expect to find the most pronounced dynamics of change in the post-industrial metropolitan cities (hypothesis 2) that have emerged as hotspots of sustainable urban lifestyle preferences [26].

Finally, we expect to observe that cohort-specific changes in everyday mobility are partly due to changes in the structural composition of the younger cohorts (and the underlying processes of changing transitions to adulthood). Therefore, controlling for educational status, labor force participation, and living arrangements should substantially reduce behavioral differences between cohorts (hypothesis 3). In other words, we expect the altered social structure or composition of Generation Y to explain some of the changes in mobility behavior.

## Data and methods

We use the MiD person-time-series dataset (*MiD-Personen-Zeitreihendatensatz*) [13], which includes the survey years 2002, 2008, and 2017. For our cohort-based analyses, we selected the 2002 and 2017 samples, which allow us to compare different birth cohorts in a cross-sectional perspective, as well as selected cohorts over different age brackets. Information on the highest educational degree and the type of household was extracted from the person datasets of 2002 and 2017, and was merged into the working dataset. For all calculations, we used the weighting factor "P_GEW".

Since our substantive interest is in examining not the prevalence of (almost) daily bicycle use,^1^ but *regular and predominant bicycle use as opposed to regular and predominant car use*, we distinguish the following categories: (1) people who use bicycles (almost) daily *and* cars at most 1–3 times a week ("predominant bicycle users"), (2) people who use cars (almost) daily *and* bicycles at most 1–3 times a week ("predominant car users"), (3) people who use *both* at most 1–3 times a week ("rare bicycle and car users"), and people who use both modes (almost) daily (“bimodal users “).

The full dataset has a sample size of n = 438,043. Our analytic sample is limited to members of the 1937–1999 birth cohorts and respondents who were between 18 and 81 years old at the time of the interviews in 2002 or 2017, respectively. After excluding the 2008 survey wave and the respondents who did not provide a valid gender statement, the sample size was reduced to n = 252,730 (Table 1).

**Table 1:** Sample selection (case numbers)

MiD person-time-series dataset (complete dataset)	438,043
Only 1937–1999 cohorts	367,841
Only persons between 18 and 81 years of age	348,692
Only 2002 and 2017 survey waves	303,998
Only persons with valid statement on mobility type	252,730

Source: Mobility in Germany: MiD person-time-series dataset (own calculations)

Based on the regional variable "RegioStaR7", we distinguish between metropolises with more than 500,000 inhabitants, a broad category of large and medium-sized cities, and rural areas. As mentioned above, we are particularly interested in the post-industrial metropolises, which we consider to be hotspots of changing everyday mobility. Based on information on the German federal states, we aggregated the cities of Berlin, Hamburg, Frankfurt, Stuttgart, Munich, and Nuremberg into the first group of post-industrial metropolises. The second group is more heterogeneous, and comprises the cities of Hannover, Bremen, Dortmund, Essen, Cologne, Düsseldorf, Dresden, und Leipzig. From a theoretical point of view, Cologne and Düsseldorf would appear to fit into the category of post-industrial metropolises. Unfortunately, the dataset does not allow for this operation. Thus, we have to keep in mind that we may be underestimating the empirical differences in mobility behavior between the post-industrial and other metropolises. The large cities/mid-sized towns category is comprised of settlements that range from large cities with fewer than 500,000 inhabitants to regional centers in rural regions. Finally, the rural areas category is comprised of small towns and villages in rural regions.

We distinguish four birth cohorts (1937–52, 1953–68, 1969–1984, and 1985–1999), which roughly correspond to the popular categories of the 68 Generation, the Baby Boomers, and Generations X and Y. Since the 2002 and 2017 surveys were conducted 15 years apart, we are able to observe the 1937–52, 1953–68, 1969–1984 cohorts at two different points in time; i.e., in two different life phases. As shown in Table 2, we map Generation X (marked in green) in young and mid-adulthood, the Baby Boomers (marked in red) in mid- and later adulthood, and the 68ers in later adulthood and retirement age. Additionally, the members of Generation Y are observed in 2017 in early adulthood (aged 18–33, highlighted in yellow). In other words, the data structure allows us to analyze behavioral changes over the life courses of the 68ers, Baby Boomers, and Generation X on the one hand, and to compare mobility behavior in early adulthood between Generations X and Y on the other.

**Table 2:**
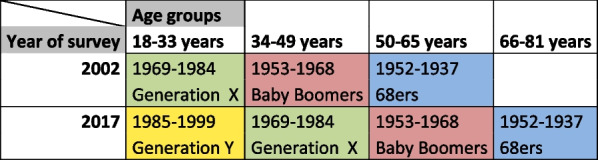
Cohorts (generations) und age groups

Table 3 presents the descriptive statistics. A comparison of the survey years reveals substantive differences in life course structures and employment patterns that could cause cohort differences in mobility behavior.

**Table 3:** Descriptive statistics

	2002	2017
Variable	Perc.	Perc.
*Mobilty type*		
(0) Rare bicycle and car use	26.9	33.4
(1) Predominant bicycle use	13.6	14.4
(2) Predominant car use	53.6	49.1
(3) Bimodal bicycle and car use	5.9	3.2
*Gender*		
(0) Men	47.6	49.4
(1) Women	52.5	50.6
Cohort		
(0) 1937–1952	30.9	22.9
(1) 1953–1968	39.4	31.1
(2) 1969–1984	29.5	25.9
(3) 1985–1999	–	18.6
Missing values	0.1	1.5
*Place of residence*		
(0) Post-industrial metropolis	9.9	11.4
(1) Other metropolis	5.3	7.3
(2) Large cities/mid-sized towns	43.7	39.1
(3) Rural areas	20.0	20.3
Missing values	21.1	21.9
*Educational status*		
(0) Lower secondary	66.4	59.7
(1) Upper secondary	32.9	36.4
Missing values	2.7	3.9
*Type of household*		
(0) Single-person	18.0	21.5
(1) Couple	31.2	36.9
(2) Three or more adults	10.8	14.0
(3) Family (children below age 18)	40.0	27.0
Missing values	–	0.6
*Employment status*		
(0) Full-time	49.6	43.5
(1) Part-time	14.7	15.3
(2) Non-employed	27.1	36.0
Missing Values	8.7	5.2
n	37,078	215,652

Source: Mobility in Germany: MiD person-time-series dataset (own calculations)

## Results

**Fig. 1:**
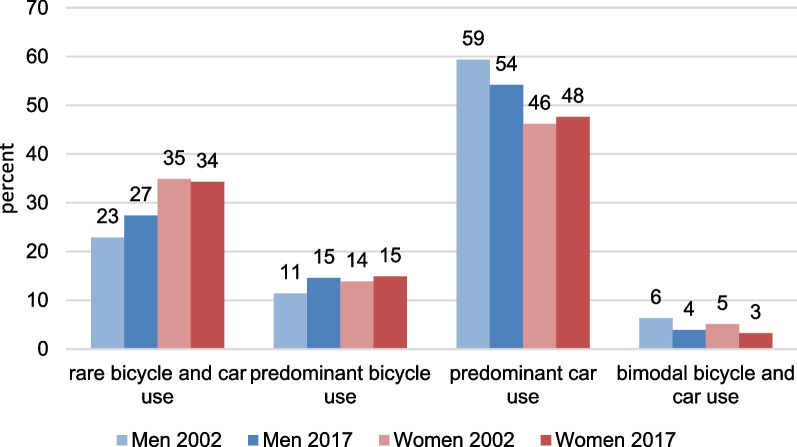
Mobility type by gender and year of survey. Source: Mobility in Germany: MiD person-time-series dataset (own calculations). Survey weights are applied

Figure 1 shows that between 2002 and 2017, the proportion of individuals who reported predominantly using a bicycle increased from 11 to 15% among men and from 14 to 15% among women. Thus, over time, gender differences in bicycle use disappeared. Overall, the indicator confirms prior findings based on day-specific measurements that reported increasing volumes and trip performance by bicycle [23].

Over the same period, the proportion of individuals who reported regularly and predominantly using a car declined from 59 to 54% among men, while it rose slightly from 46 to 48% among women. Regular bimodal transport patterns played a minor and decreasing role. Finally, more than a third of all women and more than a quarter of all men were not regularly riding a bike or driving a car.

**Fig. 2:**
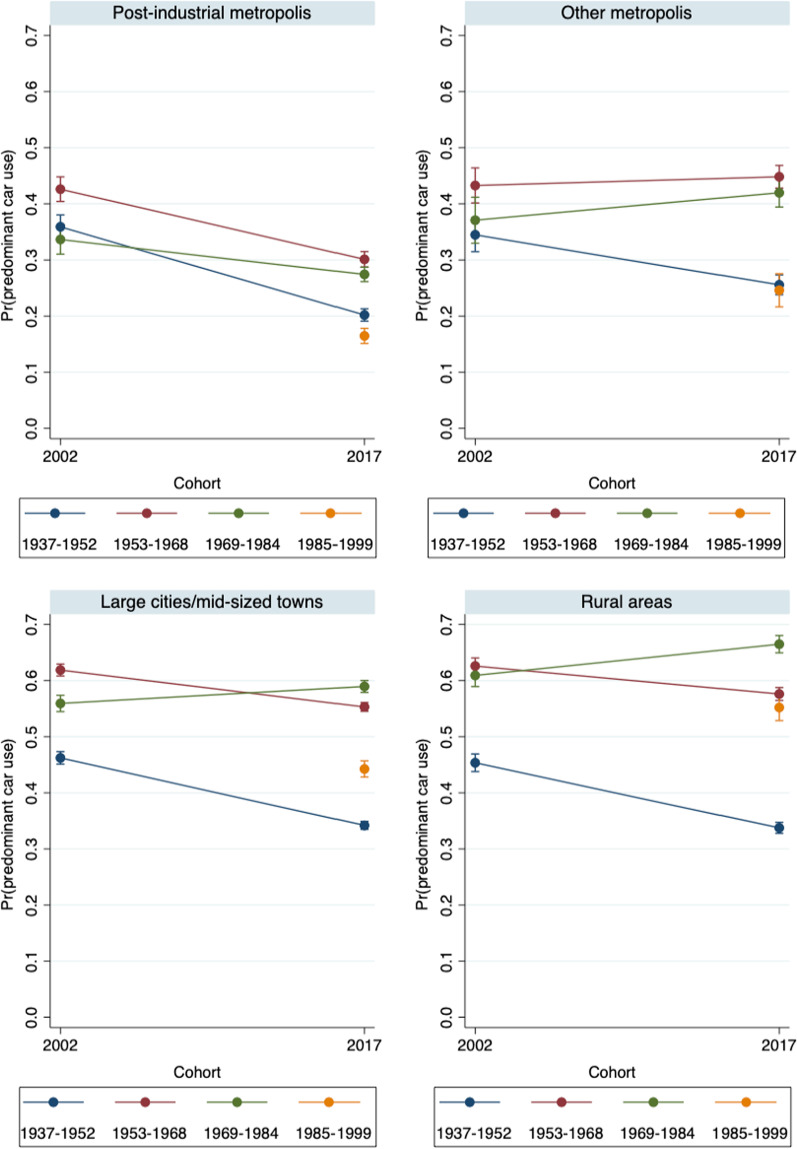
Predicted probabilities of predominant regular car use in 2002 and 2017 – by settlement type (Model 1). Source: Mobility in Germany: MiD person-time-series dataset (own calculations). *Note*: Predominant car use is defined as (almost) daily car use and bicycle use of at most 1 to 3 times per week. Survey weights are applied. See also Table 4 for main effects and standard errors

**Fig. 3:**
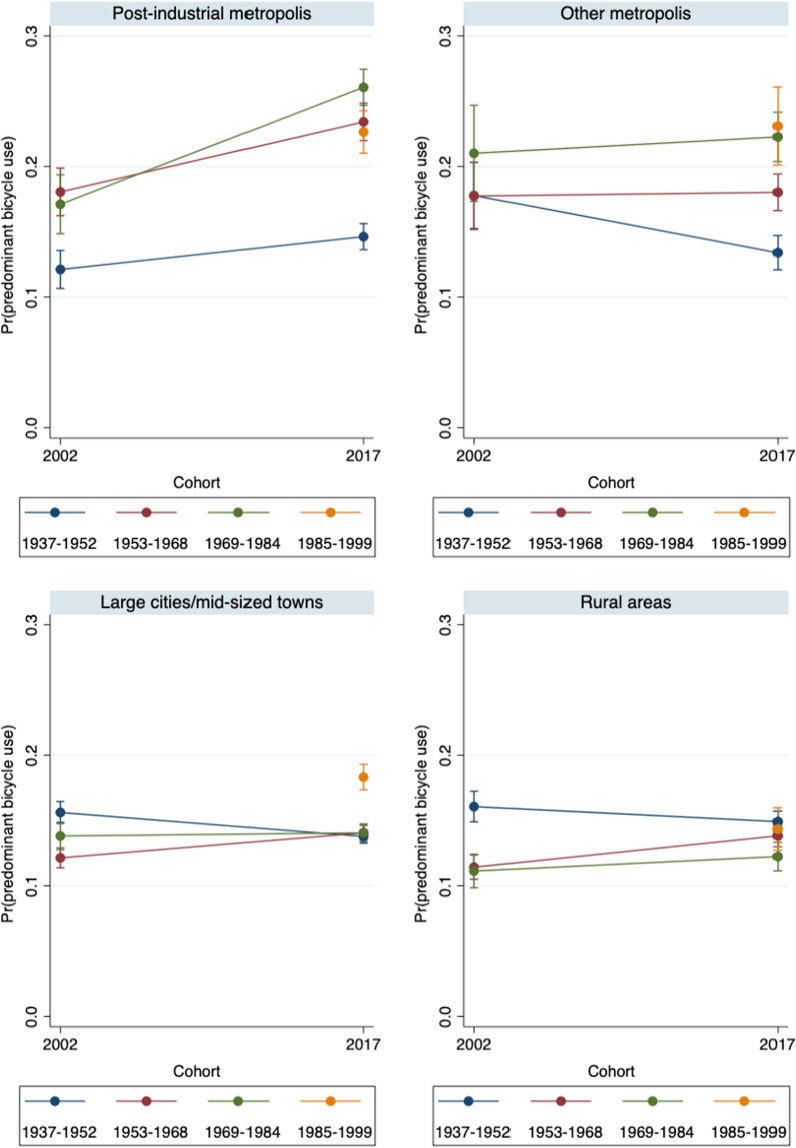
Predicted probabilities of predominant regular bicycle use in 2002 and 2017 – by settlement type (Model 1). Source: Mobility in Germany: MiD person-time-series dataset (own calculations). *Note*: Predominant bicycle use is defined as (almost) daily bicycle use and car use of at most 1 to 3 times per week. Survey weights are applied. See also Table 5 for main effects and standard errors

**Fig. 4:**
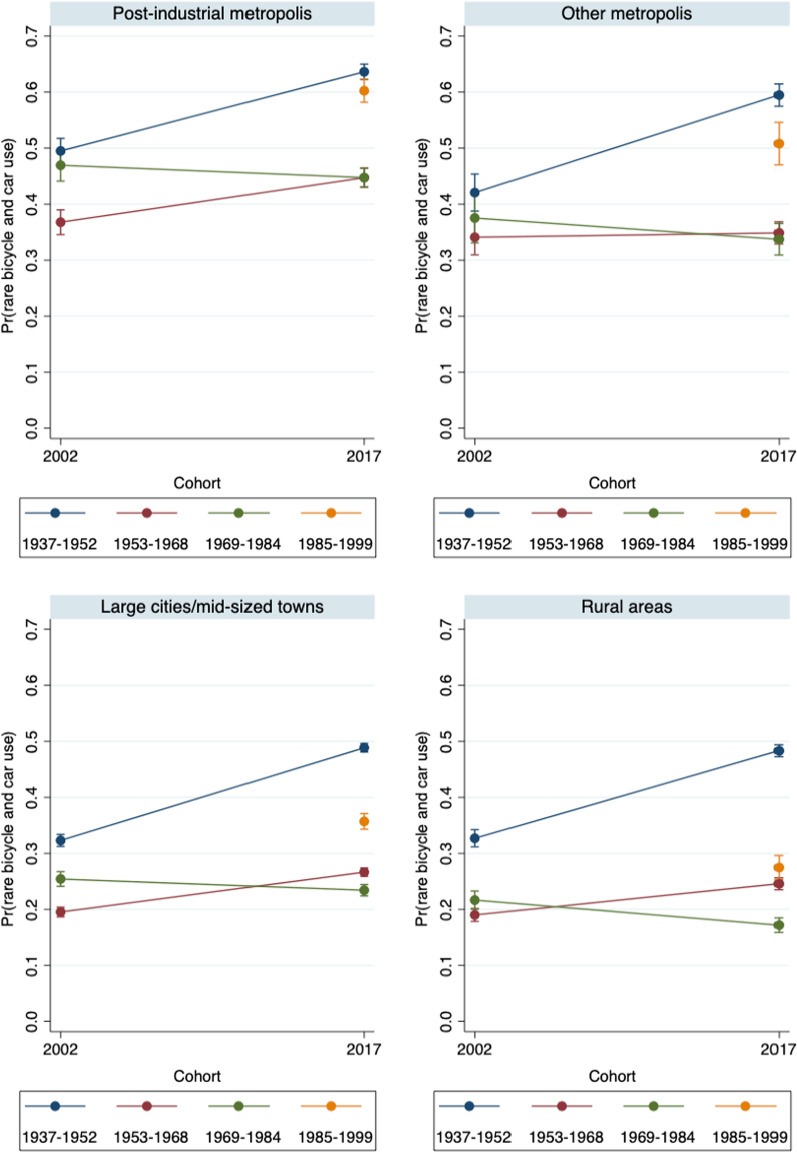
Predicted probabilities of rare bicycle and car use in 2002 and 2017 – by settlement type (Model 1). Source: Mobility in Germany: MiD person-time-series dataset (own calculations). Note: Rare bicycle and car use is defined as bicycle use of at most 1 to 3 times per week *and* car use of at most 1 to 3 times per week. Survey weights are applied. See also Table 6 for main effects and standard errors

**Fig. 5:**
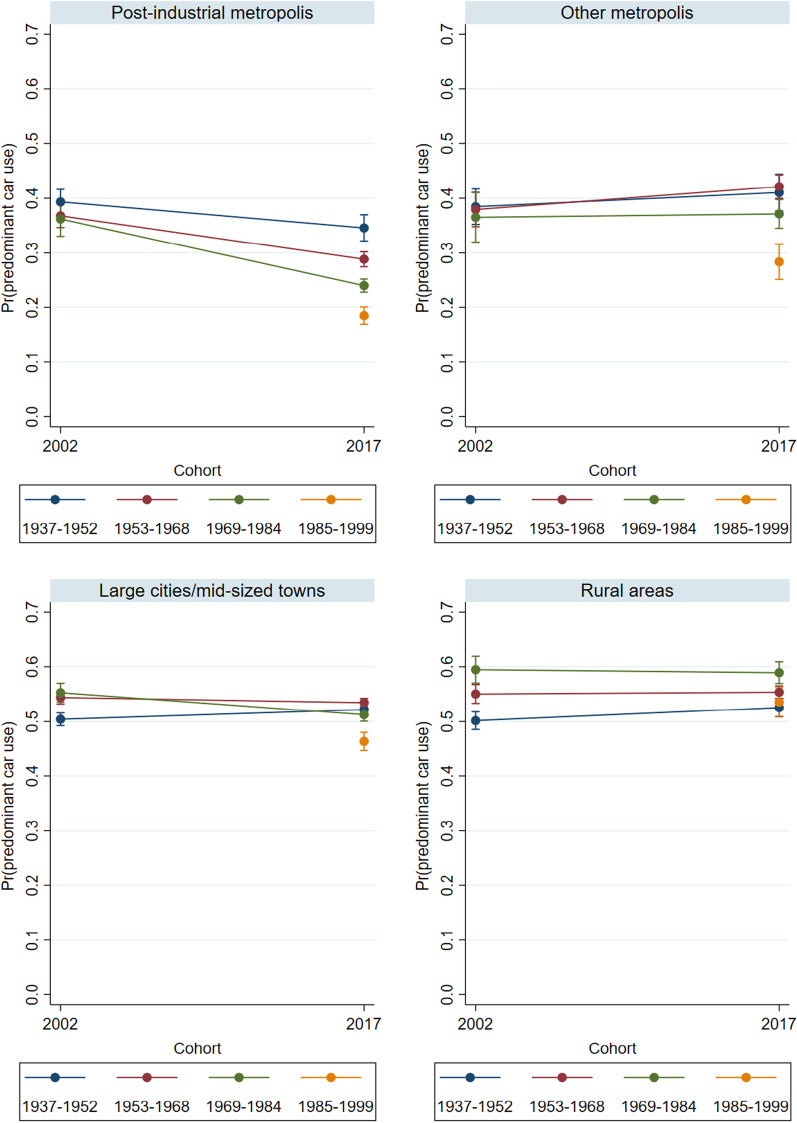
Predicted probabilities of predominant regular car use in 2002 and 2017 – by settlement type (Model 2). Source: Mobility in Germany: MiD person-time-series dataset (own calculations). *Note*: Predominant car use is defined as (almost) daily car use and bicycle use of at most 1 to 3 times per week. Survey weights are applied. See also Table 4 for main effects and standard errors

**Fig. 6:**
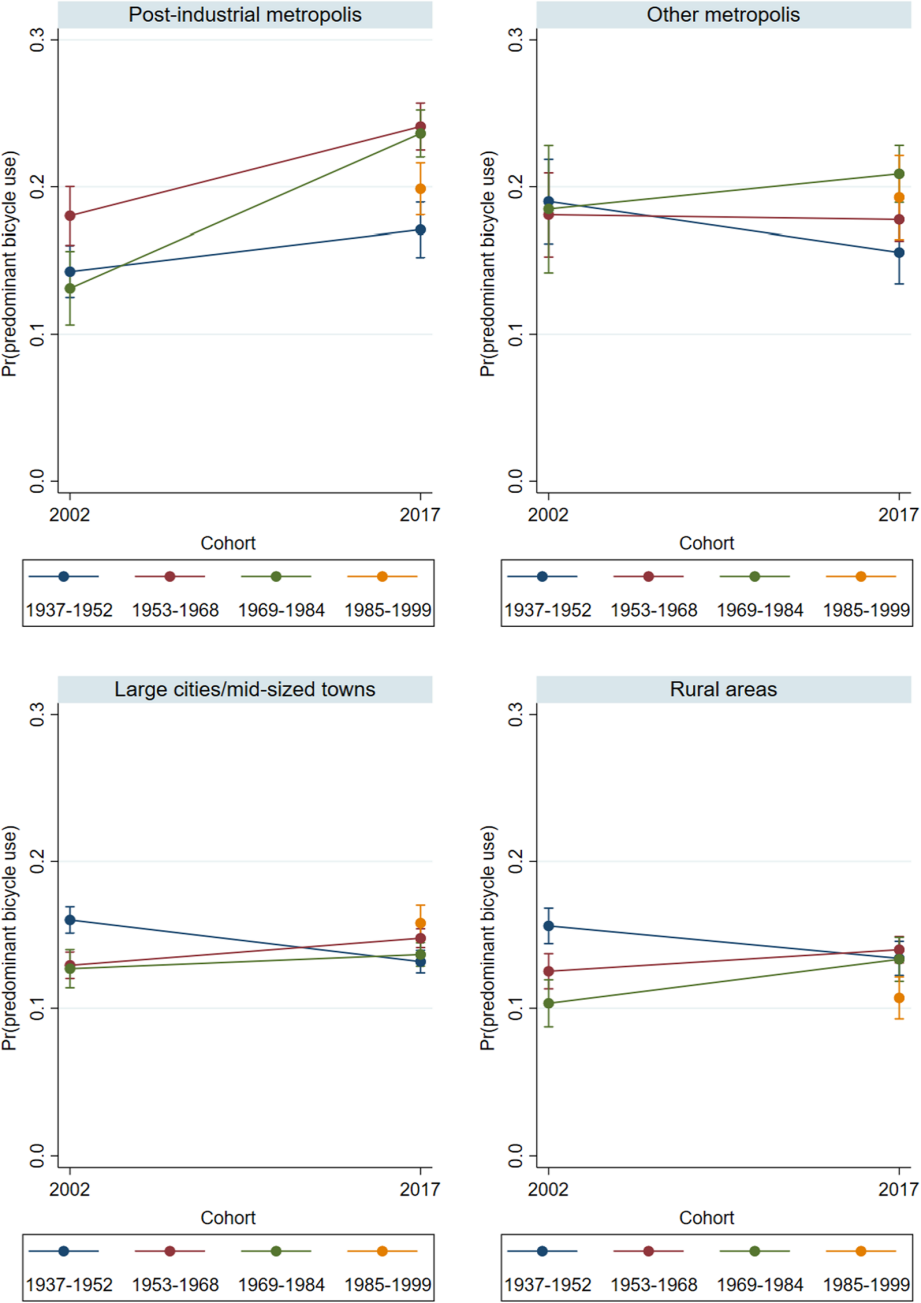
Predicted probabilities of predominant regular bicycle use in 2002 and 2017 – by settlement type (Model 2). Source: Mobility in Germany: MiD person-time-series dataset (own calculations). Note: Predominant bicycle use is defined as (almost) daily bicycle use and car use of at most 1 to 3 times per week. Survey weights are applied. See also Table 5 for main effects and standard errors

**Fig. 7:**
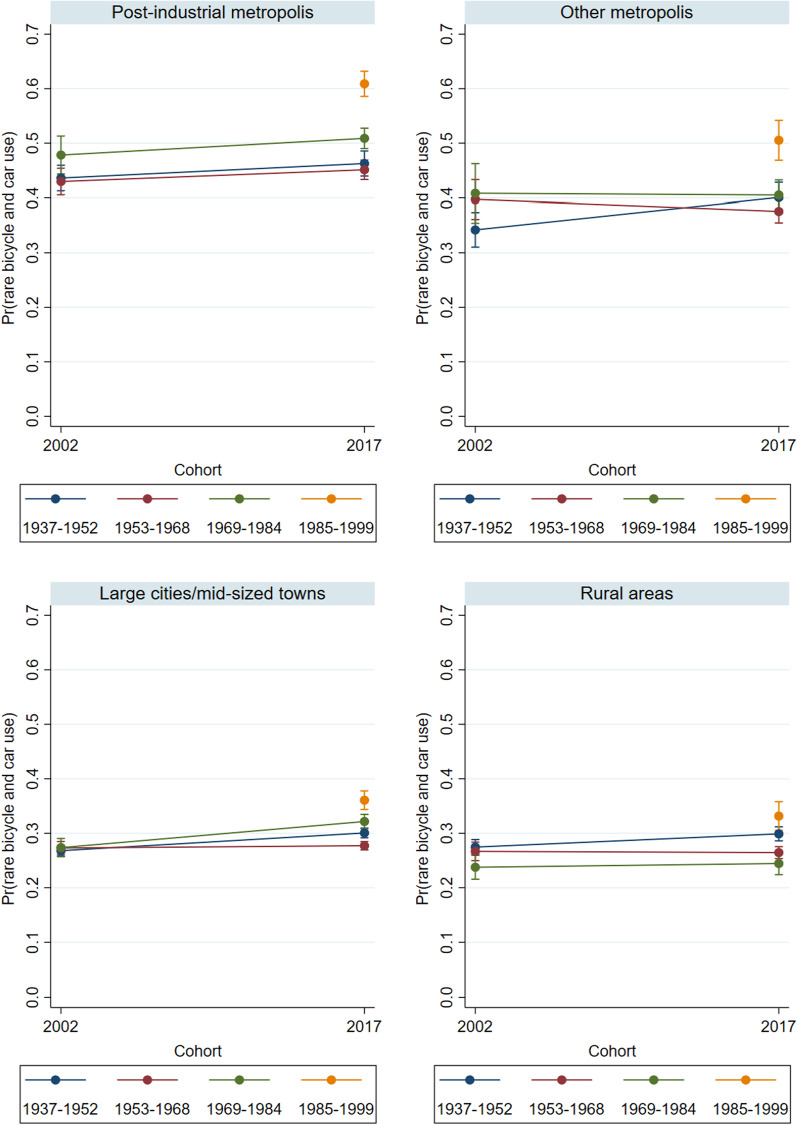
Predicted probabilities of rare bicycle and car use in 2002 and 2017 – by settlement type (Model 2). Source: Mobility in Germany: MiD person-time-series dataset (own calculations). Note: Rare bicycle and car use is defined as bicycle use of at most 1 to 3 times per week *and* car use of at most 1 to 3 times per week. Survey weights are applied. See also Table 6 for main effects and standard errors

Since the data point to converging behaviors among women and men, in the multivariate part of our study, we will skip the gender-specific analyses and control for gender instead. We expect to find that the younger cohorts (Generation Y), and especially those who are living in post-industrial metropolises, are the pioneers of changes in everyday mobility. Thus, we run multinomial logistic regressions that differentiate between the different types of everyday mobility, and present the predicted probabilities (predictive margins) of reporting predominant car use, predominant bicycle use, and rare (infrequent) car and bicycle use for our four cohorts and the four distinct settlement types in both 2002 and 2017. Technically, the analyses are based on the interaction terms of cohort, year, and settlement type. The first model (Figs. 2,3,4) only controls for gender (see Tables 4, 5, 6 in the Appendix for the main effects), while the second model additionally controls for household composition, educational attainment, and labor force participation (Figs. 5, 6, 7 and Tables 4, 5, 6).

Figure 2 displays the cohort-specific estimates of predominant car use. The lines depict differences in probabilities between survey years; i.e., cohort-specific changes over the life course. In contrast, the estimates for Generation Y in 2017 vs. Generation X in 2002 (and also for Generation X vs. Baby Boomers and Baby Boomers vs. 68ers) represent age-specific differences across cohorts.^2^

In the post-industrial metropolises, the probability of predominant everyday car use fell substantially in all cohorts between 2002 and 2017. The decline was particularly large ( − 16%) in the 1937–1952 cohort (the 68ers). In the 1953–1968 cohort (Baby Boomers), the probability of predominant car use decreased about 12 percent. Individuals born between 1969 and 1984 (Generation X) were also less likely to use a car as their predominant means of transportation ( − 7%). Finally, Generation Y had the lowest probabilities of predominant car use in 2017. Among this cohort, only 16% adhered to this pattern.

In the other settlement types, the likelihood of predominant car use changed much less. The 68er cohort was an exception, which is likely due to age-induced decreases in everyday mobility. In the “other” metropolitan cities, the Baby Boomers hardly changed their car use patterns. In the remaining two settlement types, the Baby Boomers’ car use declined, but their probability of predominant car use remained high. We observe a 62- to 55-percentage-point decline in large cities/mid-sized towns and a 62- to 58-percentage-point decline in rural areas. Among Generation X, the likelihood of predominant car use even increased, most obviously in rural areas. Finally, among Generation Y, we find a lower probability of predominant car use than among the preceding cohorts in all settlement types except rural areas. A comparison of Generation Y in 2017 and Generation X in 2002 additionally reveals a decline in the probability of predominant car use in early adulthood (age bracket 18–33).

To sum up, we find a dominant pattern of changes across cohorts in levels of predominant car use, which supports hypotheses 1a (cohort differences) and 2 (differences between settlement types). In addition, we observe that intra-cohort changes in behavioral patterns mostly occurred in the post-industrial metropolises, in which all generations became less likely to report predominant car use. In a further step, we will examine whether genuine behavioral changes occurred across cohorts and over the life course, or whether the observed changes can be attributed to socio-structural characteristics. Before we do so, we address the question of whether the changes in car use were complemented by countervailing trends in bicycle use.

Figure 3 shows the corresponding probabilities of predominant bicycle use. In the post-industrial metropolises, the predictive margins increased significantly for Baby Boomers and Generation X, resulting in probabilities of 23 and 26%, respectively, in 2017. Contrary to our expectations, Generation Y did not use bicycles more often than the older cohorts. However, a life stage-specific comparison between Generations X and Y shows that the latter were slightly more likely to use a bicycle in early adulthood. Again, the patterns in the post-industrial metropolises differed from those in the other settlement types. In the “other” metropolises, the likelihood of predominant bicycle use hardly changed over the life courses of Baby Boomers and Generation X. In 2017, the probabilities remained substantially lower than in the post-industrial metropolises (at 18 and 21%, respectively). In the other two settlement types, the probabilities of predominant bicycle use were much lower, and they hardly changed between the years. Among Generation Y, we observe higher probabilities of predominant bicycle use in the large and medium-sized cities, both cross-sectionally in 2017 and relative to those among Generation X in 2002. Thus, with respect to hypotheses 1b and 2, the empirical evidence suggests that specifically in the post-industrial metropolises, levels of predominant bicycle use increased in all cohorts, and that Generation Y did not play a pioneering role in the cycling trend.

The empirical trends in predominant bicycle use are better understood if we additionally consider the changes in rare (infrequent) bicycle and car use. Figure 4 shows that the corresponding probabilities are particularly high in the youngest cohort (and as well in the “oldest” generation). Both cohort comparisons in 2017 and age-specific comparisons between Generations X and Y suggest that rare bicycle and car use was especially widespread among Generation Y. This pattern is observed for all settlement types, but the probabilities were particularly high (60 percent) in the post-industrial metropolises.

As a next step, we will examine to what extent the observed changes in everyday mobility patterns over the life course, as well as the differences between cohorts, were driven by household composition, educational attainment, and labor force participation (see also Model 2 in Tables 4, 5, 6). Corresponding to Figs. 2, 5 shows the predicted probabilities of predominant and regular car use in 2002 and 2017. The lines now depict changes in the cohort-specific probabilities of predominant car use independent of changes in living arrangements, education, and employment; which means that the cohort-specific differences cannot be attributed to differences in these characteristics.

For the post-industrial metropolises, the results diverge from the prior findings: i.e., controlling for personal and occupational characteristics reveals a clear gradation of the probabilities of predominantly using the car in 2017. The 1937–52 cohorts now display the highest probability of predominant car use. By contrast, the Baby Boomers and Generation X both had significantly reduced probabilities of predominant car use in 2017, with the probabilities being lowest among Generation Y. A comparison of Generation Y in 2017 and Generation X in 2002 confirms that the likelihood of predominant car use was lower among the former than the latter, even after structural factors such as educational upgrading and later entry into the labor force are taken into account. In the other settlement types, we do not observe behavioral changes among the older cohorts. Thus, in the “other” metropolises and medium/large cities, the youngest cohort had lower probabilities of predominant car use than the older cohorts, but their respective probabilities remained much higher (28 and 46%) than in the post-industrial metropolises.

Taken together, the multivariate findings confirm the existence of a clear and consistent trend toward less car use in the post-industrial metropolises. After controlling for household composition, education, and labor force participation, Baby Boomers and Generation X were less likely to report predominantly using a car in 2017 than in 2002. Moreover, the probabilities of predominant car use decreased from cohort to cohort. In 2017, Generation Y also had a lower propensity to report predominant car use than other cohorts in the “other” metropolises and large/mid-sized cities. The only exception can be observed in small towns/rural areas, which displayed consistently high levels of car use among all cohorts. In sum, the findings suggest that to a large extent, Generation Y shifted away from car use in everyday mobility.

Figure 6 shows the corresponding probabilities of predominant bicycle use while controlling for education, household composition, and employment. The key finding from Fig. 3 that the youngest cohorts were not using bicycles more often than the older cohorts is confirmed. After controlling for third variables, it even appears that in the post-industrial metropolises, Baby Boomers and Generation X had a higher propensity to use a bicycle than Generation Y.

In the other settlement types, controlling for education, employment, and living arrangements confirms the initial findings that there were few changes in cohort-specific behavioral patterns. Moreover, in the category of large/mid-sized towns, Generation Y no longer exhibit increased probabilities of bicycle use. We can therefore summarize that bicycle use rose specifically among Baby Boomers and Generation X in the post-industrial metropolises, while in large/mid-sized cities and rural areas, the probabilities of predominantly using a bicycle remained much lower, and hardly differed between the cohorts.

Thus, with respect to hypotheses 1b and 2, we conclude that while predominant bicycle use became more prevalent in the post-industrial metropolises, these dynamics were not driven by the youngest cohort, but rather by Generation X and the Baby Boomers. Accordingly, the initial assumption that Generation Y pioneered alternative and sustainable modes of everyday mobility does not hold.

Finally, we look again at the probabilities of using neither a car nor a bicycle on a regular basis. Figure 1 had shown that this mobility pattern was reported by 27 percent of all men and 34 percent of all women in 2017. Figure 7 confirms the prior findings (Fig. 4) that a low propensity to use a car *and* a bike was most widespread in the post-industrial metropolises. At the same time, Generation Y stand out by displaying the highest probabilities of rare car and bicycle use. This pattern is visible in all settlement types. In general, controlling for the cohorts’ educational status, employment status, and household characteristics substantially reduced the cohort differences regarding the likelihood of reporting rare car and bicycle use. Most strikingly, for the oldest cohort, the probabilities of rare car and bicycle use became much lower, which suggests that leaving the labor market and children moving out of the household were major factors in this cohort’s mobility behavior.

With respect to our research question, the overall conclusion is that in the time period under consideration, Generation Y were more inclined than the preceding cohorts to use neither a car nor a bicycle on an (almost) daily basis.

## Conclusion

Following up on a rather general discourse on generational changes in mobility attitudes, particularly a lower affinity for cars and more sustainable value orientations among the Millennials, our paper investigated cohort-specific changes in everyday mobility in Germany. We addressed three related questions: (1) Did the members of Generation Y pioneer an increase in bicycle use, and, conversely, a decrease in car use? (2) Did these changes occur among the younger cohorts as a whole, or did they primarily affect specific subgroups, e.g., residents of large metropolises? (3) To what extent can cohort-specific patterns in mobility behavior be explained by changes in structural composition, specifically regarding education, employment status, and private living arrangements?

Our analyses of the MiD person-time-series dataset showed that between 2002 and 2017, predominant car use declined, especially in the post-industrial metropolises, and Generation Y were less attached to cars as a means of everyday mobility than prior generations. This “cohort effect” proved stable after controlling for socio-structural characteristics (hypothesis 1a). The decline in car use was most visible in the large metropolises, while it was less pronounced in other settlement types.

With respect to bicycling, the dynamics were much less clear. The Millennials did not emerge as forerunners of bicycle use (contradicting hypothesis 1b). Instead, our study showed that the increase in bicycle use over time was primarily driven by the Generation X and Baby Boomer cohorts in the post-industrial metropolises. More generally, the patterns and trends of everyday mobility varied substantially between the various settlement types (hypothesis 2). The differences in the mobility patterns between the post-industrial metropolises and the “other” metropolises also suggest that the changes in mobility behavior can be linked to more general lifestyle patterns that concentrate in specific urban environments (cf. [26, 27]. Finally, controlling for socio-structural characteristics indicated that changes in car use largely occurred irrespective of compositional factors (hypothesis 3). This finding indicates that the observed behavioral patterns may be driven by changing values and attitudes, even though we did not measure subjective orientations as such on the empirical level.

In sum, our research complemented the existing body of research on Millennials’ mobility behavior by systematically exploring age-specific patterns of car and bicycle use, while also distinguishing between birth cohorts and settlement sizes. Our analytical approach led to substantial findings regarding stability and changes in mobility behavior in Germany. Most importantly, while we did not find general cohort trends, we did observe empirical patterns that diverged across cohorts and settlement types. Moreover, changes in car and bicycle use did not appear as complementary trends. In substantive terms, the Millennial generation, despite being the least inclined to report predominant car use in the metropolises, did not emerge as “carriers” of change in everyday mobility behavior. Thus, our results may call into question simplified ideas about how more sustainable patterns of everyday mobility are generated by mechanisms of generational exchange.

Our analyses leave open issues for further research. We have to acknowledge that simultaneously differentiating aspects of bicycle and car use between cohorts, calendar years, and settlement types pushed our database to its statistical limits. The distinction between two types of metropolitan areas served as a proxy for the idea that everyday mobility is at least partly related to urban lifestyles that concentrate in “advanced” post-industrial metropolises (and possibly in specific residential areas of these cities). More fine-grained operationalization that is not primarily based on settlement size may lead to more nuanced empirical results. With regard to the role of socio-structural factors, our analyses drew on rather simple indicators that only roughly reflected the life course-specific conditions of everyday mobility. In a similar vein, the question of to what degree decreasing car use in the metropolises was actually caused by changing values and attitudes remains open. Moreover, our analyses were based on rather crude indicators of (almost) daily car and bicycle use (and a combination of the two). Thus, the finding that infrequent mobility increased across cohorts pertains to these two modes of mobility and transportation, while it remains open to what extent this trend was compensated for by public transport or pedestrian walking. Another interesting question for future research is certainly how the rapid spread of pedelecs and e-bikes will affect everyday mobility behavior. For example, the availability of e-bikes may provide older age groups and residents of suburbian and rural areas with better opportunities to use bicycles on a regular basis (cf. [46, 47]. Finally, it remains to be seen whether the COVID-19 pandemic will have lasting impacts on travel modes, especially in large towns and metropolitan areas (cf. [48, 49]).

## Data Availability

“Mobilität in Deutschland” (“Mobility in Germany”) is a nationwide survey of households on their everyday transport behavior commissioned by the German Federal Ministry of Transport and Digital Infrastructure. The dataset is available for scientific purposes via the "Clearing House Transport" of the Institute of Transport Research of the DLR (German Aerospace Center).

## Appendix

See Tables

**Table 4:** Predominant car use

	Model 1		Model 2	
*Variable*	AME	(SE)	AME	(SE)
*Year of survey*				
2002	(Ref)		(Ref)	
2017	− .0561543***	.0044848	− .0161332***	.0046927
*Gender*				
Male	(Ref)	(Ref)	(Ref)	(Ref)
Female	− .0767708***	.0043858	− .0273309***	.0047294
*Cohort*				
1937–1952	− .1914059***	.0046968	− .0183649**	.0069987
1953–1968	(Ref)	(Ref)	(Ref)	(Ref)
1969–1984	.0198468**	.0057986	− .0119407*	.006033
1985–1999	− .1043552***	.0081715	− .069835***	.0086536
*Place of residence*				
Post-industrial metropolis	− .2811143***	.0064027	-.2671929***	.0064564
Other metropolis	− .18021***	.0092427	− .1656257***	.0090995
Large cities/mid-sized towns	− .0391346***	.0055025	− .032432***	.0054203
Rural areas	(Ref)	(Ref)	(Ref)	(Ref)
*Educational status*				
Lower secondary	–	–	(Ref)	(Ref)
Higher secondary	–	–	-.040999	.0045789
*Type of household*				
Single-person	–	–	(Ref)	(Ref)
Couple	–	–	.0681526***	.0058612
Three or more adults	–	–	.0819855***	.0083893
Family (children below age 18)	–	–	.1285871***	.0073075
*Employment status*				
Full-time	–	–	.2900956***	.007319
Part-time	–	–	.1774316***	.0081302
Non-employed	–	–	(Ref)	(Ref)
*Observations*	197.225		180.884	

Source: Mobility in Germany: MiD person-time-series dataset (own calculations)

AME = Average Marginal Effects; *p < 0.05, **p < 0.01, ***p < 0.001. Survey weights are applied

4,

**Table 5:** Predominant bicycle use

	Model 1		Model 2	
*Variable*	AME	(SE)	AME	(SE)
*Year of survey*				
2002	(Ref)	(Ref)	(Ref)	(Ref)
2017	.0102555 **	.003411	.009356*	.003619
*Gender*				
Male	(Ref)	(Ref)	(Ref)	(Ref)
Female	− .0066675**	.0032729	− .0235644***	.0037676
*Cohort*				
1937–1952	− .0076619*	.0036219	− .0097003	.0056222
1953–1968	(Ref)	(Ref)	(Ref)	(Ref)
1969–1984	.0053267	.0042427	-.0046417	.0046361
1985–1999	.0300979***	.0060804	-.0053095	.0062625
*Place of residence*				
Post-industrial metropolis	.0751589***	.0056735	.0648272***	.0057546
Other metropolis	.0056735***	.0072964	.0443954***	.0068492
Large cities/mid-sized towns	.0098795*	.0040001	.0064929	.003995
Rural areas	(Ref)	(Ref)	(Ref)	(Ref)
*Educational status*				
Lower secondary	–	–	(Ref)	(Ref)
Higher secondary	–	–	.0614597***	.0035476
*Type of household*				
Single-person	–	–	(Ref)	(Ref)
Couple	–	–	− .0162474**	.0046863
Three or more adults	–	–	-.0244334	.0065399
Family (children below age 18)	–	–	-.0145085*	.0056632
*Employment status*				
Full-time	–	–	− .0340869***	.0059865
Part-time	–	–	.0271752***	.0068408
Non-employed	–	–	(Ref)	(Ref)
*Observations*	197.225		180.884	

Source: Mobility in Germany: MiD person-time-series dataset (own calculations)

AME = Average Marginal Effects; *p < 0.05, **p < 0.01, ***p < 0.001. Survey weights are applied

5,

**Table 6:** Rare bicycle and car use

	Model 1		Model 2	
Variable	AME	(SE)	AME	(SE)
*Year of survey*				
2002	(Ref)	(Ref)	(Ref)	(Ref)
2017	.0661566 ***	.0042783	.0233452***	.0045056
*Gender*				
Male	(Ref)	(Ref)	(Ref)	(Ref)
Female	.0906985***	.0044004	.0627137***	.0045845
*Cohort*				
1937–1952	.2063783	.0048565	.0230829***	.0065019
1953–1968	(Ref)	(Ref)	(Ref)	(Ref)
1969–1984	− .0216083***	.0056116	.024755***	.0063724
1985–1999	.032835***	.0051375	.0935052***	.008961
*Place of residence*				
Post-industrial metropolis	.2294762***	.0070944	.2241316***	.0070975
Other metropolis	.1419963***	.0099082	.1352556***	.0089728
Large cities/mid-sized towns	.0340138***	.0051765	.0289853***	.0050202
Rural areas	(Ref)	(Ref)	(Ref)	(Ref)
*Educational status*				
Lower secondary	–	–	(Ref)	(Ref)
Higher secondary	–	–	− .0254109***	.004533
*Type of household*				
Single-person	–	–	(Ref)	(Ref)
Couple	–	–	− .0579703***	.0058538
Three or more adults	–	–	− .0712658***	.0086894
Family (children below age 18)	–	–	− .1378613***	.0074987
*Employment status*				
Full-time	–	–	− .2582026***	.0077771
Part-time	–	–	− .2251945***	.0083785
Non-employed	–	–	(Ref)	(Ref)
*Observations*	197.225		180.884	

Source: Mobility in Germany: MiD person-time-series dataset (own calculations)

AME = Average Marginal Effects; * p < 0.05, ** p < 0.01, *** p < 0.001. Survey weights are applied

6.

## Footnotes

1 In the 2017 sample, bicycles also included e-bikes and pedelecs. Six percent of the respondents reported having an e-bike/pedelec available.

2 For all estimates, 83% confidence intervals are plotted. We follow Austin and Hux [45]: 195, who recommend constructing 83% rather than 95% confidence intervals when seeking to assess “whether or not two means are significantly different from one another at the α = 0.05 level.”.
